# Different Effects of Cigarette Smoke, Heated Tobacco Product and E-Cigarette Vapour on Orbital Fibroblasts in Graves’ Orbitopathy; a Study by Real Time Cell Electronic Sensing

**DOI:** 10.3390/molecules27093001

**Published:** 2022-05-07

**Authors:** Janos K. Aranyosi, Erika Galgoczi, Annamaria Erdei, Monika Katko, Mariann Fodor, Zoltan Ujhelyi, Ildiko Bacskay, Endre V. Nagy, Bernadett Ujhelyi

**Affiliations:** 1 Department of Ophthalmology, Faculty of Medicine, University of Debrecen, Nagyerdei Körút 98, 4032 Debrecen, Hungary; ifj.dr.aranyosi.janos@med.unideb.hu (J.K.A.); mfodor@med.unideb.hu (M.F.); 2 Doctoral School of Clinical Sciences, University of Debrecen, Nagyerdei Körút 98, 4032 Debrecen, Hungary; 3 Divison of Endocrinology, Department of Internal Medicine, Faculty of Medicine, University of Debrecen, Nagyerdei Körút 98, 4032 Debrecen, Hungary; galgoczi.erika@med.unideb.hu (E.G.); erdei.annamaria@med.unideb.hu (A.E.); katko.monika@med.unideb.hu (M.K.); nagy@internal.unideb.hu (E.V.N.); 4 Department of Pharmaceutical Technology, Faculty of Pharmacy, University of Debrecen, Nagyerdei Körút 98, 4032 Debrecen, Hungary; ujhelyi.zoltan@pharm.unideb.hu (Z.U.); bacskay.ildiko@pharm.unideb.hu (I.B.); 5 Institute of Healthcare Industry, University of Debrecen, Nagyerdei Körút 98, 4032 Debrecen, Hungary

**Keywords:** retrobulbar fibroblast, Graves orbitopathy (GO), cigarette smoke extract, alternative tobacco products, real time cell electronic sensing (RT CES)

## Abstract

Thyroid autoimmunity in Graves’ disease (GD) is accompanied by Graves’ orbitopathy (GO) in 40% of the cases. Orbital fibroblasts (OF) play a key role in the pathogenesis and cigarette smoking is a known deteriorating factor. Alongside conventional cigarettes (CC) new alternatives became available for smokers, including heated tobacco products (HTP) and E-cigarettes (ECIG). We aimed to study the cellular effects of smoke extracts (SE) in orbital fibroblasts. Primary OF cultures from GO and NON-GO orbits were exposed to different concentrations of SE (1%, 50%) and the changes were followed using Real Time Cell Electronic Sensing (RT-CES). Untreated GO and NON-GO cells had different maximum cell index (CI) values of 3.3 and 2.79 respectively (*p* < 0.0001). CC, HTP and ECIG treated NON-GO fibroblasts exhibited peak CIs of 2.62, 3.32 and 3.41 while treated GO cells’ CIs were higher, 5.38, 6.25 and 6.33, respectively (*p* < 0.0001). The metabolic activity (MTT) decreased (*p* < 0.001) and hyaluronan production doubled (*p* < 0.02) after 50% of CC SE treatment in all cell cultures. GO fibroblasts were more sensitive to low concentration SE then NON-GO fibroblasts (*p* < 0.0001). The studied SEs exerted different effects. RT-CES is a sensitive technique to detect the effects of very low concentration of SE on fibroblasts.

## 1. Introduction

Graves’ disease (GD) is an autoimmune thyroid disease, that results in hyperthyroidism and affects up to 2% of the population [1]. In approximately 40% of the cases GD is accompanied by Graves orbitopathy (GO), being the most common extrathyroidal manifestation of GD [2]. Swollen, retracted eyelids and proptosis are caused by the orbital autoimmune process, which leads to enlargement of the extraocular muscles and increased volume of the orbital connective tissue [3,4]. The impaired function of extraocular muscles leads to diplopia and the inadequate tear film causes dry eyes that can lead to corneal ulceration. The most severe form is dysthyroid optic neuropathy (DON) with progressive loss of visual acuity and permanent damage or loss of visual function [5].

Orbital fibroblasts (OF) play a key role in the pathogenesis due to the expression of autoantigens [4,6]. Fibroblast activation is followed by connective tissue remodeling with lymphocyte infiltration and cytokine-dependent activation; extracellular matrix (ECM) expansion contributes to the pathogenesis of GO [7]. Hyaluronan (HA) is the major glycosaminoglycan of the ECM and has an important role in cell-ECM and cell-cell interactions. Due to its hydrophilic nature, HA contributes to the edema of the connective tissue in GO [3,4].

The well described negative influence of smoking on disease course [8] is comprised of reactive oxygen species (ROS) generation, enhancement of effects of oxidative stress, intensified adipogenesis and enhanced HA production of OFs [9,10]. Smokers have a greater chance of developing GO, and the severity of the disease correlates positively to the number of smoked cigarettes [11]. Tobacco smoke is a mixture of nearly 7000 chemical compounds, some of which are in the gas phase, such as carbon monoxide (CO) or aldehydes and some remain in the particulate phase, including polycyclic aromatic hydrocarbons, nicotine and other alkaloids [12]. Sixty nine components were proven to be carcinogens and are present in both mainstream and side stream cigarette smoke [13]. Smoking is known to be a major predisposing factor for pulmonary [14] and non-pulmonary cancers [15,16], as well as the greatest environmental risk factor for GD and GO [17].

In the recent years, alternatives of conventional cigarettes (CC) were introduced to the public. Electronic cigarettes (ECIG) and heated tobacco products (HTP) have entered the market. They were created to deliver nicotine through an inhaled aerosol, without generating smoke by combusting tobacco. Since these products generate less side stream emissions than CC in indoor environments, are more socially acceptable [18]. Analytical studies have identified toxic substances in the ECIG aerosol that are considered carcinogenic, such as nitrosamines, polycyclic aromatic hydrocarbons (PAH), free radicals and toxic gases, but in substantially lower concentrations than in CC smoke [19]. Similarly, reduced amounts of harmful constituents were detected in the HTP aerosol compared to CC smoke, presumably due to the lower temperatures [20,21]. Meanwhile the nicotine levels appeared to be only slightly lower than in CC smoke [22]. Besides the chemical composition, the physical properties of the aerosol also have a great impact on the biological outcome of the exposure. Ultrafine particles (<100 nm) are toxicologically important, because of higher deposition in the respiratory tract. Second hand emissions of HTP and ECIG products contain lower levels of ultrafine products than CC. Several in vitro studies focusing on the cytotoxic, oxidative and inflammatory effects of HTP and ECIG products have been published recently [18,19,23]. However, it is difficult to interpret the results, given the diversity of ECIG and HTP products, complexity of ingredients and the different type of target cells. The majority of studies using airway epithelial cells showed that ECIG and HTP aerosol can facilitate cytotoxicity and the production of pro-inflammatory mediators, but to a smaller degree compared to CC [24,25,26].

There is limited information regarding the effects of these alternative smoking products on orbital fibroblasts and their function. To the best of our knowledge, ECIG and HTP products have not been examined in this respect. In the present study we compare the effects of ECIG, HTP vapors and CC in NON-GO and GO orbital fibroblast cultures. We show that real time cell electronic sensing is a sensitive technique to detect early changes evoked by smoke exposure.

## 2. Results

### 2.1. High Concentration Smoke Extract Exposure of Fibroblasts

#### RT-CES

**Figure 1:**
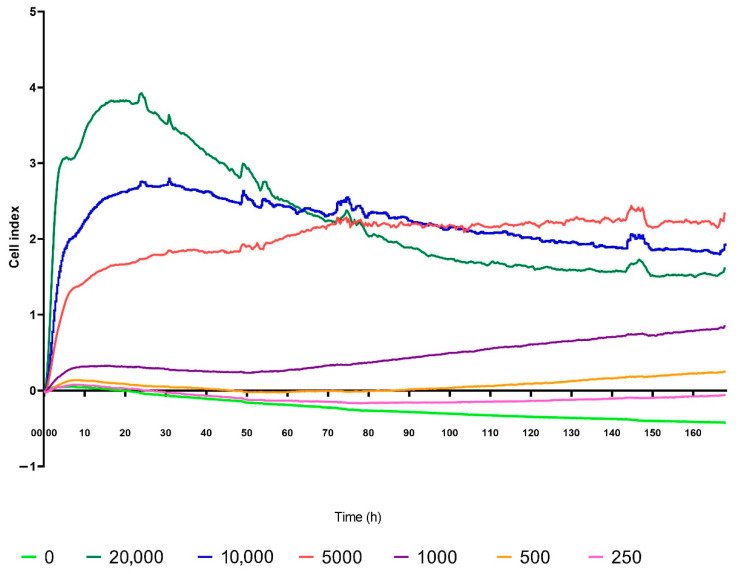
Identification of the optimum cell density for RT-CES measurements. The cells were seeded 250, 500, 1000, 5000, 10,000 and 20,000 per well. In this 168-h experiment, the 10,000 cells/well density was found optimal (blue curve). All experiments were performed in triplicates.

First, we used high CC concentration (50%). In our experiments, we aimed to see if HA secretion and metabolic activity of OFs is related to CI measured by real time cell electronic sensing. For the identification of optimum cell density, first we used the RT-CES system to monitor the CI curves at six different OF densities. Based on this measurement (Figure 1), the cell number per well was set at 10,000 in 200 µL for all subsequent experiments.

**Figure 2:**
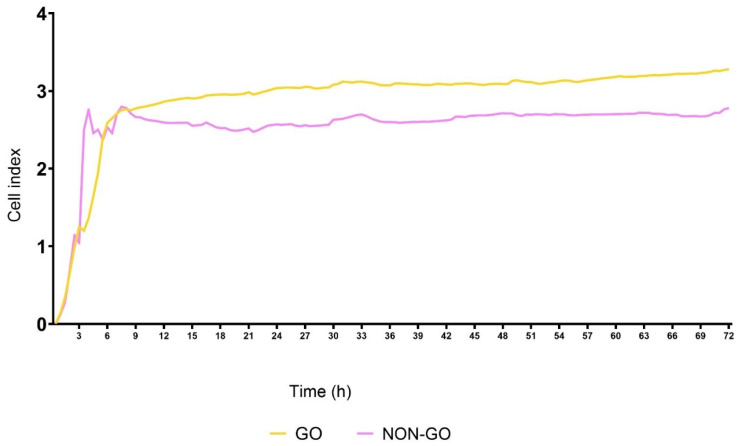
Comparison of the basal cell index values. The cell index of untreated GO cells was 3.3 and 2.79 of untreated NON-GO cells at the selected density (10,000 per well). All experiments were performed in triplicates.

**Figure 3:**
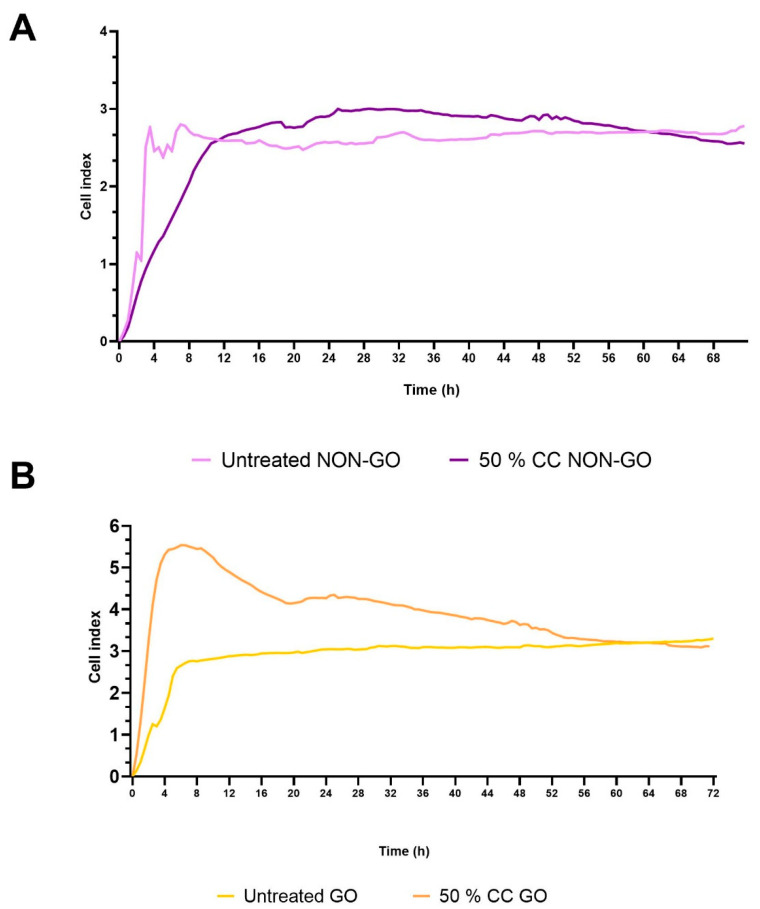
The effect of 50% conventional cigarette extract on the cell index using RT-CES. (**A**) NON-GO OF, (**B**) GO OF cells treated with a high concentration (50%) of CC showed an increase in the CI of GO cells but not in NON-GO cells, compared with the untreated cells. All experiments were performed in triplicates.

The basal cell index values of the GO cells differed significantly from NON-GO cells (*p* < 0.0001, Figure 2). The origin of the cells determined their behavior, they responded differently to the treatment (*p* < 0.0001). As seen in Figure 3A, the NON-GO OFs did not respond to the 50% CC (*p* = 0.46); the CI of NON-GO OFs did not increase after adherence, and the treatment did not cause increase in the CI levels. The maximum of CI (CI = 3.00) was reached at 29 h. The GO OFs behaved differently; as shown in Figure 3B the cell adhesion took about 4–6 h, and the CI of treated GO OFs increased significantly compared to untreated GO fibroblasts (*p* < 0.0001), reaching its maximum at 6 h (CI = 5.53). After this, a rapid decline was observed, CI values decreased to the level of untreated GO OFs at 60 h.

### 2.2. Metabolic Activity

**Figure 4:**
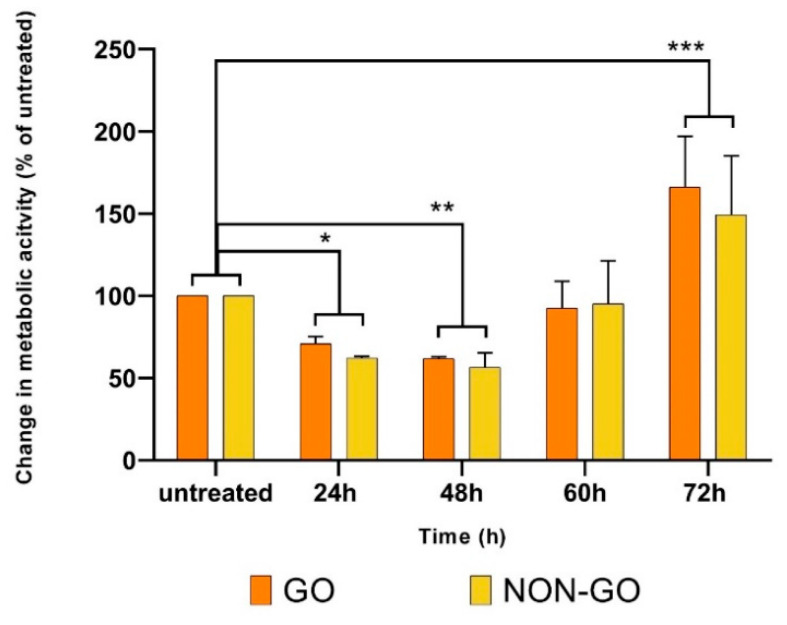
The effect of 50% CC smoke extract on the metabolic activity of GO and NON-GO fibroblasts There was no difference in effect based on the origin of the cells. All experiments were performed in triplicates. Data were analyzed using repeated measures ANOVA followed Tukey’s multiple comparisons post hoc test; the 100% means the untreated cells metabolic activity, and the result shown as % of untreated (mean ± SD) * *p* < 0.03, ** *p* < 0.02, *** *p* < 0.005.

With regard to metabolic activity (Figure 4), the GO and NON-GO cells behaved differently. At the 24th and 48th hours of the treatment, the metabolic activity of CC treated cells was significantly lower compared to the untreated cells (both GO 24 h *p* < 0.03, 48 h *p* < 0.01 and NON-GO 24 h *p* < 0.011, 48 h *p* < 0.01.) There was no difference in the effect of CC treatment on the metabolic activity between the 24 h and 48 h time interval (*p* = 0.07). At 60 h the difference in the metabolic activity between the CC treated and untreated cells disappeared in all cell lines (GO *p* = 0.66, NON-GO *p* = 0.89). After 72 h the CC treated lines had significantly higher activity rates, than the untreated cells (GO *p* = 0.0013, NON-GO *p* = 0.0041).

### 2.3. Hyaluronan Production

**Figure 5:**
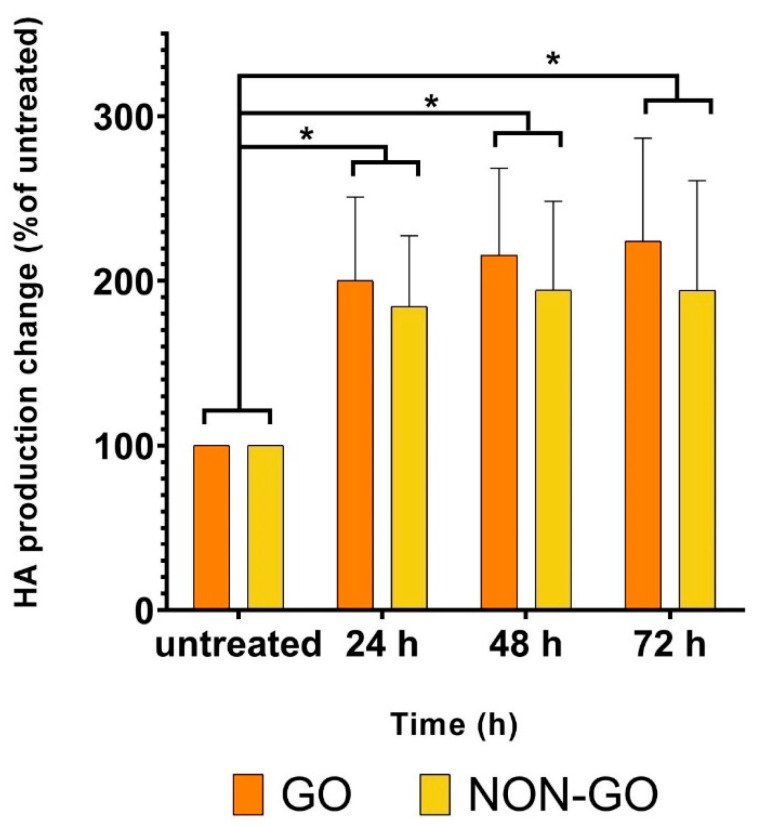
Effect of 50 % CC smoke extract on hyaluronan secretion of GO and NON-GO fibroblasts. All experiments were performed in triplicates. Data were analyzed using repeated measures ANOVA followed Tukey’s multiple comparisons post hoc test. The 100% means the untreated cells hyaluronan production, and the result shown as % of untreated (mean ± SD) * *p* < 0.02.

CC treatment caused an increase in the HA production of both NON-GO and GO cell lines in every measured time point (GO *p* < 0.002, NON-GO *p* < 0.04). The GO and NON-GO cells responded to 50 % concentration CC treatment with a constant 2-fold HA production (Figure 5). The HA production was not different in GO and NON-GO fibroblasts, neither in untreated, nor in treated cultures (untreated *p* = 0.99, 24 h *p* = 0.95, 48 h *p* = 0.87, 72 h *p* = 0.67).

### 2.4. Low Concentration Smoke Extract Exposure of Fibroblasts

#### RT-CES

In the next set of experiments all GO and NON-GO cultures were exposed to low concentration (1%) SE, which equals to approximately 8 cigarettes per day. In low concentration, CC, and both HTP and ECIG extracts were examined in all OF cultures (Table 1).

**Figure 6:**
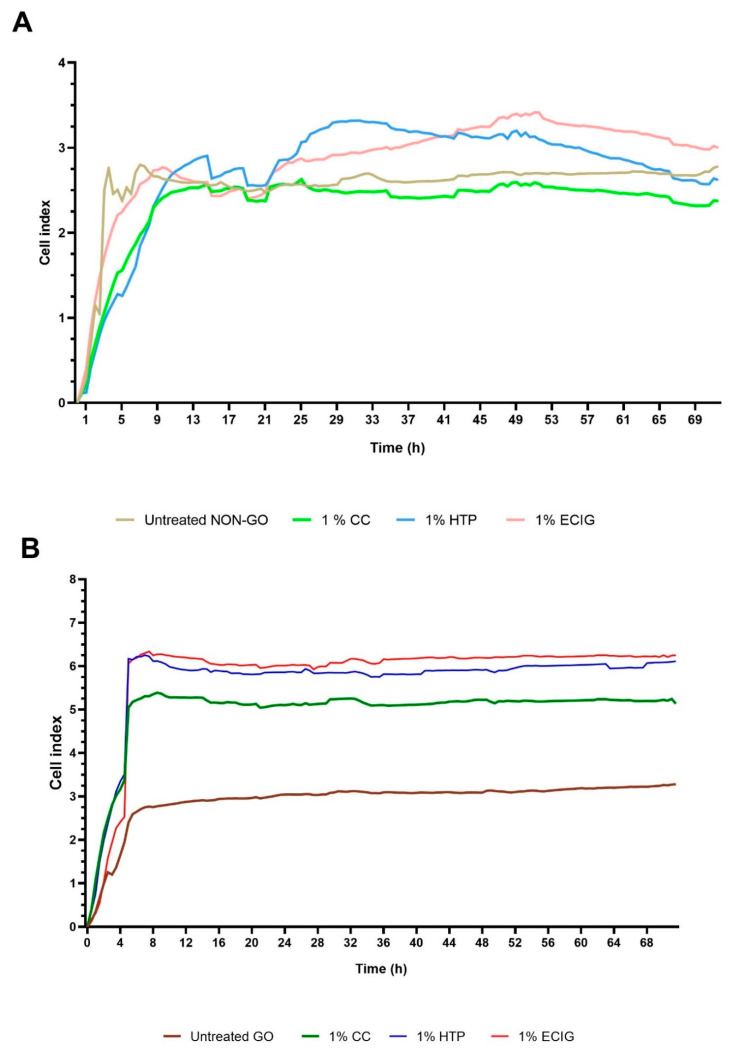
The effect of 1% CC, HTP and ECIG on the cell index of NON-GO OF (**A**) and GO OF (**B**). All experiments were performed in triplicates. All tobacco product treatments caused significant elevation in cell index in GO OFs, while NON-GO OFs were less affected by either treatment.

Untreated NON-GO cells (Figure 6A) showed a fast increase, reaching 2.5 CI values after 4 h, followed by a short declining period before reaching their peak after 8 h (CI = 2.79). From this point, their curve was mainly flat, values alternating between 2.47 and 2.78. After CC treatment, the same NON-GO cells had similar values as the untreated ones with the highest CI values of 2.62. In comparison, NON-GO cells treated with HTP and ECIG showed significant differences in the CI (*p* < 0.0001); both HTP and ECIG treated NON-GO fibroblasts acted similarly until the 22nd hour, when the CI started to increase in both lines. The HTP NON-GO line reached its maximum at 32 h (CI = 3.32), and the ECIG line at 52 h (CI = 3.41).

The untreated GO cells showed a flat curve after adhesion, CI values alternating between 2.75 and 3.3 (Figure 6B). Markedly different curves were seen after SE exposure. After adhesion at 5.5 h, CC treated GO CI values also followed a mainly flat curve, peaking at 9 h (CI = 5.38); HTP and ECIG GO cells exhibited similar behavior having plateau-like curves, but reaching higher values after adhesion compared to CC treated cells. ECIG treated GO cells peaked at 7.5 h (CI = 6.33) and the HTP GO lines peaked at 7 h (CI = 6.25). The CIs in response to CC, HTP and ECIG were statistically different (*p* < 0.0001).

### 2.5. Metabolic Activity

**Figure 7:**
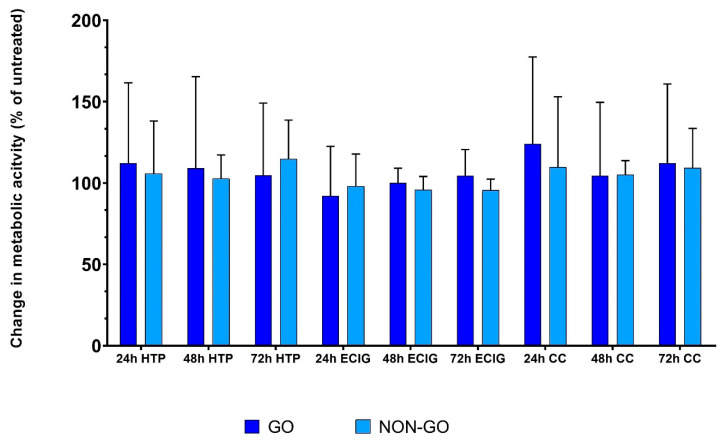
Metabolic activity of GO and NON-GO OF after 1% HTP, ECIG and CC treatment. There was no difference in the metabolic activity of the cell lines after these treatments. The result are shown as % of untreated (mean ± SD). The data were analyzed using repeated measures ANOVA followed Tukey’s multiple comparisons post hoc test. All experiments were performed in triplicates.

After 24, 48 and 72 h of treatment with 1% HTP, ECIG and CC, there was no significant difference in metabolic activity in any cell line after any of the treatment compared to untreated cells (Figure 7). The basal metabolic activity of untreated GO and NON-GO cells were also not different. In contrast to 50% CC treatment (see Figure 4 above), the 1% of HTP, ECIG and CC had no effect on metabolic activity (*p* = 0.9010). 

### 2.6. Hyaluronan Production

**Figure 8:**
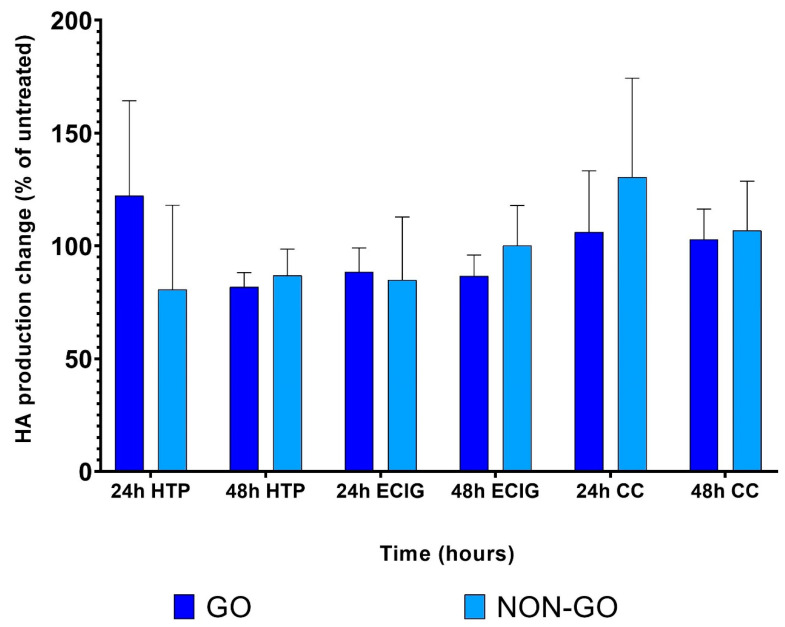
The effect of 1% HTP, ECIG and CC on hyaluronan production of GO and NON-GO cells. The results are shown as % of untreated (mean ± SD). Data were analyzed using paired *t*-test. All experiments were performed in triplicates.

We did not find any difference between the HA production of treated and untreated cells. All of the cell lines behaved similarly in this concept, irrespective of the site of origin (*p* = 0.8302). The 1% SE had no effect on the examined cells neither 24 h, nor 48 h after treatment (*p* = 0.139) (Figure 8).

## 3. Discussion

OFs are targets of immunological processes in Graves’ disease, their normal functions being dysregulated by autoimmune mechanisms [27]. OFs secrete large amounts of HA in the presence of various cytokines [27] and can differentiate to mature adipocytes, known to express the thyrotropin receptor [28,29]. The cellular changes lead to the characteristic clinical appearance of GO patients, involving the enlargement of extraocular muscles and expansion of orbital connective tissue. When compared with fibroblasts from other sites, OFs show an increased inflammatory reaction to various stimuli [30].

Smoking is a major risk factor in the development of GO, and there is a positive correlation between the severity of GO and the number of cigarettes smoked per day [9]. Alternatives to traditional smoking have been introduced recently; ECIG and HTP are popular replacements. However, it is unknown whether ECIG or HTP have different effects on the course of GO.

RT-CES has found several applications in cell biology, including cell proliferation and spreading [31,32], wound healing [33], cytotoxicity [34,35,36] and cancer research [37]. It is a technique that is used to calculate cell index, an estimation of cell proliferation and metabolic activity, and is frequently used on immortalized cell lines [34,38]. However, it has rarely been applied on other cell types, such as fibroblasts [39,40]. The advantage of this technique is the time dependent setting, which enabled us to analyze the effect of smoke extract in orbital fibroblasts.

The objective of this study was to examine the cellular response of OFs in vitro to smoke exposure from conventional cigarettes and recently available tobacco products. Human primary OF cultures were obtained from patients with and without GO. To the best of our knowledge, this is the first study to examine the effects of CC, HTP and ECIG products on OFs derived from NON-GO and GO patients and the first time utilization of real-time cell electronic sensing on orbital fibroblasts.

In the first set of experiments we examined the effect of conventional cigarette smoke extract in high concentration (50%) on GO and NON-GO OFs.

NON-GO OFs did not respond to the 50% CS treatment (*p* = 0.46) (Figure 3A); however, GO OFs behaved differently (Figure 3B). By RT-CES, the CI of treated GO OFs increased significantly compared to untreated GO OFs (*p* < 0.0001).

However in this set of experiments we used higher concentration of CS extracts described earlier [9], the results were unexpected and suggest more different effect on fibroblast cell than toxicity itself.

Previous results have all examined shorter durations on a scale of 0.5–24 h (3–10% [41], 1–5% [10], 10–50% [42]. High toxicity was also shown at concentrations of 50% [42] in endothelial cells. The changes observed during the first 72 h in our study suggest, that those fibroblast cells that survived the toxicity of high concentration treatment underwent changes which affect GO fibroblasts differently than controls.

We analyzed the results from RT-CES and compared to that of the MTT assay to evaluate metabolic activity. HA production of treated and untreated OFs were also analyzed. MTT and HA assays were performed after 24, 48 and 72 h, while RT-CES provided continuous data for 72 h. At 24th and 48th hours of the treatment, the metabolic activity of CC treated cells was significantly lower compared to the untreated cells (both GO and NON-GO, 24 h *p* < 0.001, 48 h *p* < 0.0001). At 60th hours the difference in the metabolic activity between the CC treated and untreated cells disappeared in all cell lines (GO *p* = 0.3, NON-GO *p* = 0.63). After 72 h the CS treated lines had significantly higher metabolic activity rates, than the untreated cells. (GO *p* = 0.007, NON-GO *p* = 0.012) We hypothesize that, the increase in metabolic activity on treated cells seen at 72 h is due to a breakdown of toxic compounds of CC in the medium which allowed the cell number to increase. Proliferation is dependent on cell density in OFs [43]. The GO and NON-GO cells responded to 50% concentration CC treatment with a constant 2-fold HA production (Figure 5), which is in accordance with the negative effect of smoking on the course of the disease.

By adding 50% CC to the cells, GO and NON-GO cells behaved differently concerning the CI, but we did not find any difference in metabolic activity and HA production rates between GO and NON-GO cells with or without CC treatment. In contrast, the CI of these cell lines depend on the origin of the cells (Figure 2). In case of the CC GO OF, the fast growth in CI, then the following continuous decrease can relate to the toxic effect of the high dose CC. This could have caused cell death and detachment from the electrodes, leading to changes in CI. The same toxic effect could have caused the decrease in the MTT assay in the first 24 h. By the 72nd hour the metabolic activity was greater in the CC treated GO and NON-GO OF lines.

As high (50%) concentration may have been toxic and diminished the metabolic activity of the fibroblasts, in the second set of experiments low (1%) concentration has been used mimicking smoking of 8 cigarettes per day. Indeed, 1% CC smoke extract had negligible effect on NON-GO OFs, yet we hypothesize that it was not toxic in this concentration.

RT-CES detected a remarkable difference in the effect of 1% HTP, ECIG and CC in OFs. Differential responses were seen according to OF origin: GO OFs were more sensitive to smoke exposure, and this sensitivity was dependent on the type of smoke used. However, each tested SE evoked obvious cell index increase in all cell cultures. Although, when treated with 1% HTP, ECIG and CC, neither GO nor NON-GO fibroblasts showed different metabolic activity on MTT assay or hyaluronan production, clearly showing the unique sensitivity of the RT-CES technique.

According to the RT-CES results (Figure 6) ECIG caused the highest CI change on GO OF. HTP had lower effects on CI, and the CC treated cells showed the least response to treatment in the GO group. Untreated GO cells were in the mid-range, showing smaller CI compared to the treated groups. In the case of NON-GO cells the 1% HTP, ECIG and CC caused a lower difference in the CI, but it was still significant (Figure 6A). Each of them had smaller CI values compared to treated GO lines, and their activity was similar.

While the CI values show time-dependent, dynamic changes in cell activity at 1% CC, HTP and ECIG concentration, no significant effect on metabolic activity and HA production was seen.

One limitation of our study is the use of a relatively new method, the RT-CES. Further, it is unclear how we can translate our findings to everyday practice. There are multiple systemic and local factors present simultaneously in vivo in the orbit, including immune and endocrine processes, increased pressure, and the unique tissue structure. Fibroblasts are only one of the targets of the pathogenetic process.

In conclusion, the three different smoke extracts influenced the CI, measured by RT-CES, of GO and NON-GO OFs in low concentrations which failed to evoke changes in HA secretion or metabolic activity. High concentration CC smoke extract had affected only GO fibroblast behavior by RT CES; however, HA production was elevated in both GO and NON-GO fibroblasts. RT-CES is a sensitive technique to detect smoke extract evoked fibroblast changes. As HTP and ECIG smoke had different effect on GO OFs than CC smoke, it can be assumed that new ways of smoking might affect Graves’ disease patients differently regarding GO. Our approach is a suitable in vitro model to investigate the behavior of orbital fibroblasts under different conditions.

## 4. Materials and Methods

This in vitro study was performed on four GO orbital fibroblast cell lines from GO patients, and four control orbital fibroblast cell lines that were derived from patients who underwent enucleation due to intraocular tumor, with no history of thyroid disease. First, high concentration (50%) of CC extract was added to the cells, in order to compare its effects measured by Real Time Cell Electronic Sensing (RT-CES), as well as in HA synthesis and cell metabolic activity assays. In the next set of measurements, a lower concentration (1%) of smoke extracts (SE) of the three different SEs, CC, HTP and ECIG was used. All measurements were time dependent, as RT-CES provides continuous readings for 168 h; HA and MTT assays were performed after 24, 48 and 72 h. All measurementswere repeated three times, the end results being the average of the triplicate measurements.

### 4.1. Tissue Samples and Cell Cultures

Human orbital tissues were obtained during orbital decompression surgeries of GO patients (samples 1–4), and enucleations in patients with no history for thyroid disease (NON-GO controls, sample 5–8). Patients’ data are summarized in Table 2. Enucleation was performed because of intraocular tumors in all four cases, limited to the eyeball, without scleral invasion; the extraocular space and tissues were intact. All patients provided written consent. The study was approved by the National and Institutional Ethics Committee in accordance with the Declaration of Helsinki (5913/2012/EKU (84/13)).

The cell cultures were established by the method of Bahn et al. [44]. Briefly, small tissue pieces were put into culture dishes containing Medium 199 (M199) with Earle’s salts with 20% (*v*/*v*) FBS and penicillin–streptomycin, and cultured at 37 °C, 5% CO_2_, in a humidified incubator, and the medium was changed every 3–4 days. After removing the tissue pieces, cell cultures were maintained in M199 with 10% (*v*/*v*) FBS. Then the cells were stored in freezing medium in liquid nitrogen until used. Human primary orbital fibroblasts (OF) were studied at low passage numbers (2 to 8).

### 4.2. Generation of Smoke Extract

The preparation of smoke extracts (SE) was performed using a previously described method by Cawood et al. [9]. Four reference 1R6F cigarettes, containing 8.6 mg tar and 0.7 mg nicotine per piece were smoked through 30 mL of puffer using a specific pump device (MILLIPORE XF54 230-50), Only 75% of the length of cigarettes was burnt, producing 350 mL of smoke-air mixture. The solution was sterilized via 0.20 µm membrane filters. According to Bernhard et al., a 12.5% solution of CC equals 100 cigarettes smoked daily [45]. We used a lower and higher concentration (1%, 50%) of CC in the study.

The vapors of two non-conventional smoking devices were also tested. Using a commercially available HTP device (iQOS, Philip Morris International Global Services Inc., New York, NY, USA), four tobacco sticks of regular flavour were used to create SE, (12 puffs/stick, 48 puffs total), as previously described by Zagoriti et al. [23]. The ECIG device (Eleaf iStick Pico, Shenzhen, China) was also commercially available. The power was set to 16 W and we used the liquid provided by the manufacturer, containing propylene glycol (49.4%), glycerol (49.4%), and nicotine (1.2%) [23]. We collected 48 puffs of aerosol. Although only the product of HTP device creates smoke, while ECIG devices do not produce smoke in the classic meaning, this solution will also be referred to as SE in the subsequent sections. For dilutions, M199 was used to achieve the specified culture concentrations. The untreated cell lines also received solvent control at the same time as treatment was added to treated cell lines.

### 4.3. Real-Time Cell Electronic Sensing Technique (RT-CES)

To evaluate the effect of prepared SEs on cell behavior, RT-CES measurements had been performed. Although this is a relatively novel method of label-free cell monitoring, its reliability is based on several previous publications [31,34]. The device provides real-time data by measuring changes in electric impedance on the golden electrodes located at the bottom of the 16 well plates. Without cells, the ions of the culture media can fluctuate freely on the electrodes, i.e., the gold-covered button of the wells. As cells adhere, they cover the electrodes, modifying the ion flow [46]. The system uses alternating current; hence it measures the electric impedance. The cells in M199 with or without SE have been added to the wells. Real-time data have been collected which provide accurate account of cell behavior. Cell Index (CI) is the ratio of the change in impedance compared to the impedance measured at the start of the experiment. All cell lines were measured for 168 h, however after 72 h no changes were detected on any of them and for this reason, and for better interpretation we decided to present data from the first 72 h of the experiment. Data was collected every 15 min. After launching the examination, the cells required a few hours to adhere to the plate surface; data could be processed afterwards.

### 4.4. Cell Metabolic Activity Assay 

MTT assay was used to determine the metabolic activity of cultured cells. Cells were plated in 96-well plates at a density of 10^4^ cells/well and allowed to grow in a CO_2_ incubator at 37 °C for 7 days in culture medium with SE or with only vehicle. The supernatants were removed and stored at −20 °C until the experiments were performed. At the first three days the MTT was added to the cells after 21th, 45th and 69th hour after plated. Briefly, 10 µL MTT (5 mg/mL)/100 µL culture medium was added. Then the cells were incubated for 3 h in a CO_2_ incubator at 37 °C. The cells depending on their metabolic activity can convert the yellow MTT into water-insoluble blue formazan. The blue formazan crystals were dissolved in dimethyl-sulfoxid (DMSO). The absorbance at 595 nm was detected using a Beckman Coulter, DTX 880 Multimode Detector (Beckman Coulter Inc., Brea, CA, USA). Cell metabolic activity was expressed as the percentage of the untreated control.

### 4.5. Quantitation of Hyaluronan

The secreted HA in the supernatant was measured using the DuoSet Hyaluronan Kit (R&D Systems, Minneapolis, MN, USA), according to the manufacturer’s instructions. In each case, results were adjusted for the HA content of FBS. HA production was expressed as the percentage of the untreated control.

### 4.6. Statistical Analysis

Statistical analysis was performed for RT-CES results using repeated measures ANOVA followed Tukey’s multiple comparisons post hoc test (GraphPad Prism 8.0.1, GraphPad Software, San Diego, CA, USA). The level of statistical significance was set at *p* < 0.05.

## Figures and Tables

**Table 1:** CI values measured on all cell lines displayed in specific time points.

	50% CC NON-GO	50% CC GO	1% CC GO	1% HTP GO	1% ECIG GO	1% CC NON-GO	1% HTP NON-GO	1% ECIG NON-GO	Untreated GO	Untreated NON-GO
8 h	2.05	5.44	5.30	6.11	6.24	2.04	2.08	2.63	2.75	2.79
16 h	2.76	4.41	5.15	5.88	6.02	2.48	2.67	2.43	2.55	2.90
24 h	2.90	4.26	5.09	5.86	6.01	2.57	2.88	2.82	2.55	3.00
32 h	2.99	4.14	5.24	5.84	6.12	2.47	3.31	2.93	2.64	3.12
48 h	2.90	3.69	5.20	5.91	6.19	2.52	3.15	3.34	2.69	3.08
60 h	2.71	3.23	5.20	6.02	6.22	2.49	2.89	3.21	2.69	3.16
72 h	2.56	3.11	5.24	6.11	6.24	2.37	2.62	3.01	2.71	3.26

**Table 2:** Origin of primary orbital fibroblasts.

**GO Patients**					
**Samples**	**Patients’ Age**	**Gender**	**Operation Type**	**GO Stage at Operation**	**Diagnosis**
Sample 1	37 years	female	Orbitotomy, lipectomy	inactive	GO
Sample 2	44 years	female	Orbitotomy, lipectomy	inactive	GO
Sample 3	49 years	male	Orbitotomy, lipectomy	inactive	GO
Sample 4	42 years	female	Orbitotomy, lipectomy	inactive	GO
**Control Patients**					
**Samples**	**Patients’ Age**	**Gender**	**Operation Type**	**GO Stage at Operation**	**Diagnosis**
Sample 5	66 years	male	Enucleation	-	Malignant choroidal melanoma
Sample 6	71 years	male	Enucleation	-	Malignant choroidal melanoma
Sample 7	69 years	male	Enucleation	-	Malignant choroidal melanoma
Sample 8	38 years	male	Enucleation	-	Malignant choroidal melanoma

## Data Availability

Data is available on request from the authors.

## Sample Availability

Not available.
